# Improving isolate recovery and identification of the Shiga toxin type in Shiga toxin nucleic acid test-positive feces

**DOI:** 10.1128/jcm.00429-26

**Published:** 2026-08-14

**Authors:** Byron M. Berenger, Thomas Griener, Linda Chui

**Affiliations:** 1 Microbiology, Alberta Precision Laboratories 590576, Calgary, Alberta, Canada; 2 Provincial Laboratory for Public Health, Alberta Precision Laboratories 590576, Calgary, Alberta, Canada; 3 Department of Pathology and Laboratory Medicine, Cumming School of Medicine, University of Calgary 70401, Calgary, Alberta, Canada; 4 Department of Laboratory Medicine and Pathology, University of Alberta 3158, Edmonton, Alberta, Canada; Mayo Clinic, Rochester, Minnesota, USA

**Keywords:** Shiga toxin-producing *E. coli *(STEC), polymerase chain reaction, culture, feces, humans, molecular diagnostic techniques, bacteriological techniques, culture media, gastroenteritis, cycle threshold value

## Abstract

Infections caused by Shiga toxin-producing *Escherichia coli* (STEC) strains carrying Shiga toxin 2 (*stx*2) are more likely to result in severe complications; however, most nucleic acid amplification tests used for STEC diagnosis do not differentiate between *stx*1 and *stx*2. We therefore sought to optimize *stx* typing and isolate recovery methods to guide clinical and public health management. *stx* polymerase chain reaction (PCR)-positive feces were cultured using CHROMagar STEC and gram-negative broth, with Stx1 and/or Stx2 antigen detection by enzyme immunoassay (EIA) on colony growth or turbid broth. When cultures were EIA-negative, growth from MacConkey agar (MAC), Trypticase soy broth, and the gram-negative broth was then tested using a lab-developed typing PCR for *stx*1 and *stx*2. Colonies were isolated on CHROMagar STEC or MAC and identified using the typing PCR. Using both EIA and typing PCR, the *stx* types were identified in 96.0% of cases (381/397). In 65.2% (259/397) of cases, culture was EIA-positive, of which 34.0% (87/256) were Stx2-positive. Among cultures that were EIA-negative but typing PCR-positive, 64.8% (79/122) were *stx*2-positive (*P* < 0.0001 compared to EIA-positive). Using both EIA and typing PCR resulted in 72.6% (286/394) of cases with successful attempts at isolate recovery, compared to 60.7% (239/394) with EIA alone. *E. coli* O157 was recovered from more EIA-positive cases (19.3%, 46/239) than EIA-negative ones (4.3%, 2/47) (*P* = 0.0097). Typing PCR on cultures improves *stx* typing (particularly *stx*2) and isolate detection compared to EIA alone. Screening BD Max PCR and subsequent typing PCR results showed excellent concordance.

**IMPORTANCE:**

*Escherichia coli* strains with one or both types of Shiga toxins (*stx1* and *stx2*) are a common cause of bacterial diarrhea and can lead to serious complications such as kidney failure, especially in children. Infection by *stx2*-positive strains is more likely to do so. Therefore, knowing whether the infection is caused by a strain carrying *stx2* is important for risk assessment and case follow-up. The conventional way to diagnose these infections is to grow the bacteria from stool, but most laboratories currently use nucleic acid detection (e.g., bacterial DNA detection by polymerase chain reaction [PCR]), and these assays do not differentiate between the two toxin genes. Culture is therefore required to determine toxin type, as well as for public health outbreak investigations, which require an isolate for whole-genome sequencing for serotyping and cluster analysis. We identified culture media and a PCR-based method to detect *stx2* in culture that improved the detection of *stx2* and isolate recovery. Our findings provide more accurate results for clinicians to improve patient care and tools for public health teams to control and prevent outbreaks.

## INTRODUCTION

Shiga toxin-producing *Escherichia coli* (STEC) is one of the most common causes of food-borne bacterial diarrheal illness (1, 2). Notably, it can cause hemolytic uremic syndrome (HUS), especially in children, where the incidence varies from 4 to 20% (3). Pathogenicity is primarily driven by the phage-borne, plasmid-encoded toxins Shiga toxin (Stx) 1 and 2. The presence of STEC strains with the *stx*2 gene is much more likely to cause severe disease and extraintestinal complications (3). Serotype O157 represents 15%–40% that cause human disease, of which almost all produce Stx1 and Stx2 (1, 3, 4). Only approximately 30% of non-O157 strains have the *stx*2 gene (4).

Rapid detection of STEC and specific identification of *stx*2 strains is necessary for optimal clinical management and public health investigation (3, 5). Antibiotics increase the risk of developing HUS, and a timely diagnosis of STEC ensures avoidance or discontinuation of antibiotics. Furthermore, infections with *stx*2 strains warrant closer clinical follow-up with more frequent laboratory testing to detect early manifestations of HUS. There is also evidence that early hydration therapy in children may prevent HUS (3, 6, 7). Additionally, in our jurisdiction, Public Health investigators use the presence of *stx*2 to initiate a more intensive or rapid investigation.

Culture-independent diagnostic tests (CIDTs), such as polymerase chain reaction (PCR), have reduced the time to STEC results by several days and improved case detection rates. In 2023, at US FoodNet surveillance sites, more than 99% of STEC diagnoses were made by CIDT (1), a trend paralleled in many jurisdictions worldwide (8–10). Unfortunately, most US Food and Drug Administration (FDA) and Health Canada-approved commercial molecular assays used for the diagnosis of bacterial diarrhea do not differentiate between *stx*1 or *stx*2. Therefore, the determination of the *stx* type is dependent on other methods such as culture.

In addition to allowing determination of the *stx* type(s) present, culturing positive specimens should be performed because it aids public health responses. First, when managing cases with a sensitive occupation or attending childcare facilities, only culture results are accepted by public health authorities as pre-exclusion criteria in many jurisdictions ([8, 11]; and as outlined in many notifiable disease guidelines available online. Second, culture to recover isolates allows for whole-genome sequencing and serotyping of isolates, which are necessary for surveillance and for cluster analysis as an early signal of an outbreak and to track outbreaks.

Despite these fundamental requirements to culture CIDT-positive feces, only 80%–88% were cultured at US FoodNet sites (2016–2018 and 2021) (12). In the same catchment area in 2023, only 57% of cases yielded an isolate (1). Including an expanded catchment area, culture was positive in only 42.4% (see Table 1 in reference 1). Culture growth, and subsequent recovery of an isolate, from CIDT-positive feces varies widely depending on the media and process used to identify growth or isolates (20%–76%, Table S1) (1, 9, 13–21). Many of these methods use enzyme immunoassays (EIAs) to identify culture-positive samples and to retrieve an isolate. More successful methods require a combination of different culture media and picking of multiple colonies identified using PCR. Culture methods employed in high-volume diagnostic laboratories, such as frontline laboratories in our province, are typically designed to balance the sensitivity and specificity required in the setting of a high-volume lab, staffing limitations, and lack of specific expertise available at more focused public health/reference laboratories. In 2025, the Association of Public Health Laboratories (APHL) STEC working group identified these gaps and called for studies to investigate the role of enrichment broths and determine the specificity and sensitivity of different media for identifying and isolating STEC (5).

For the diagnosis of bacterial gastroenteritis, our laboratory uses a commercially available multiplex PCR assay (BD Enteric Bacterial Panel), which includes the detection of *stx* genes. We subsequently use a lateral flow EIA (*SHIGA TOXIN QUIK CHEK*) on agar or broth growth to identify the Stx subtype(s) present, determine specimen culture positivity for purposes of public health case investigation/management, and to select specimens to be sent to the public health lab for isolate retrieval and typing. Based on the above algorithm and isolate recovery rates of only 55%–60%, we sought to improve upon this standard methodology. Our primary goal was to improve the identification of the *stx* types present and, secondarily, to increase the recovery of an isolate for serotyping and whole-genome sequencing. Cases with EIA-negative cultures were cultured onto additional media, then a lab-developed *stx* typing PCR was performed on the cultures to determine the *stx* gene status and the most suitable media for isolate recovery.

## MATERIALS AND METHODS

Our study included all feces specimens submitted to the Alberta Precision Laboratories diagnostic microbiology laboratory in Calgary, AB, Canada, from November 2022 to May 2024 (except for 29 August to 30 September 2023, due to a large O157 outbreak), for investigation of bacterial gastroenteritis as part of routine clinical care. Only the first positive sample for an individual was included in the study. Specimens were submitted without transport media and stored at 4°C for testing within 24 h and tested using the BD Enteric Bacterial Panel on the BD Max instrument (Becton Dickinson, Mississauga, Ontario, Canada), a US FDA (moderate complexity) and Health Canada-approved multiplex PCR. The BD Max detects the presence of the *stx* genes without differentiating between *stx*1 and *stx*2. Other targets included are *Salmonella* spp.*, Campylobacter jejuni, Campylobacter coli,* and *Shigella*/Enteroinvasive *E. coli*.

BD Max PCR was tested according to the manufacturer’s instructions. Cycle threshold (Ct) values, as determined by the instrument, were documented for each positive sample. As an additional quality review of *stx*-positive results, amplification curves were reviewed by a medical laboratory technologist, and specimens for cases with minimal exponential rise and irregular patterns were repeated. Such samples were included in the study only if they were positive on repeat. Otherwise, they were excluded along with the *stx* PCR-negative samples.

All feces positive for *stx* by BD Max PCR were set up for culture the following day. Separate loops of feces were plated on a CHROMagar STEC (CHROMagar, Saint-Denis, France) and into 8 mL of gram-negative broth (GN broth; Dalynn Biologicals, Inc., Calgary, AB, Canada). Agar and broth were incubated for 18–24 h at 35°C ± 2°C. Using the *SHIGA TOXIN QUIK CHEK* lateral flow enzyme EIA, the presence of Stx 1 and/or 2 antigens was detected from a sweep of mauve colonies as per the manufacturer’s product insert (TechLab, Inc., Blacksburg, VA). Mauve colonies are presumptive of STEC on CHROMagar STEC. If mauve colonies were absent or the mauve colonies from CHROMagar STEC were EIA-negative, the EIA was performed on the GN broth (as per the product insert). A previous evaluation of performing *QUIK CHEK* on GN broth demonstrated improved detection of Shiga toxin types and better isolate recovery rate by using GN broth as compared to CHROMagar STEC agar alone (22).

**Fig 1:**
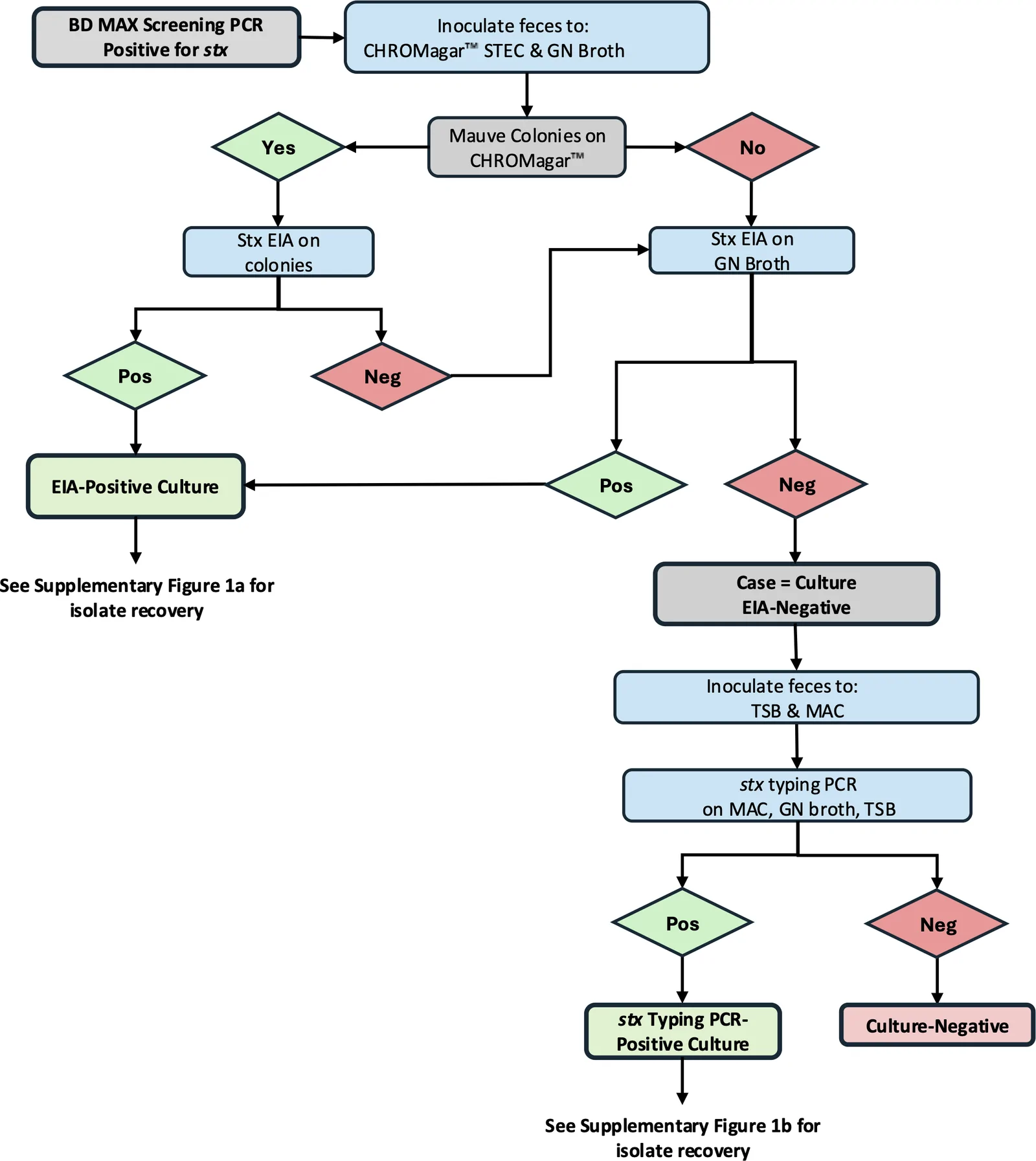
Processes used to identify Shiga toxins 1 and/or 2 antigen (Stx) or genes (*stx*) in culture using the SHIGA TOXIN QUIK CHEK EIA or lab-developed *stx* typing PCR, respectively. GN broth = gram-negative broth; STEC = Shiga toxin (Stx)-producing *E. coli*; Pos = positive; Neg = negative; MAC = MacConkey agar plate; TSB = trypticase soy broth.

For all cases that were culture EIA-positive, the GN broth and a sweep of the CHROMagar colonies (if the colonies were EIA-positive) were sent to the Alberta Provincial Public Health Laboratory (ProvLab) for isolate recovery and whole-genome sequencing (Fig. 1). Isolates were sub-cultured onto a CHROMagar STEC and MacConkey (MAC; Dalynn) agar. If mauve colonies were present on the CHROMagar STEC, they were sub-cultured onto blood agar plates, and O157 agglutination (BD Difco *E. coli* O Antiserum O157, Becton Dickinson) was performed. If negative, a lab-developed *stx* typing PCR that differentiates between *stx1* and *stx2* (23) was performed on colony sweeps from both types of agar plates and on three mauve colonies from the CHROMagar STEC plate. If the *stx* typing PCR of the mauve CHROMagar colonies was negative, up to 15 colonies (different morphotypes when present) were picked from the MAC agar for individual colony *stx* typing PCR. If sweeps from both agar plates were negative, the GN broth was sub-cultured onto MAC agar, and 15 colonies were picked as described above.

For BD Max *stx-*positive but reflex culture EIA-negative cases, GN broth and feces were sent to the ProvLab within 24 h of the EIA results for additional culture and *stx* typing PCR. Feces were stored at 4°C for 24–72 h after receipt at ProvLab before being plated onto MAC agar and into TSB (Dalynn), which were incubated for 18–24 h at 35°C ± 2°C. The *stx* typing PCR (23) was performed on a sweep of the MAC agar growth, the TSB, as well as the GN broth from the diagnostic microbiology laboratory. If the sweep of colonies from the MAC agar was *stx* typing PCR-positive, an isolate was sought by performing PCR on 15 lactose-fermenting colonies from the MAC agar. If the direct MAC agar was negative, whichever primary broth had a *stx* typing PCR Ct value of 32 or less was subbed to MAC agar, and 15 colonies were picked for *stx* typing PCR as described above.

To perform the *stx* typing PCR, an individual colony was emulsified into 100 µL of rapid lysis buffer (100 mM NaCl, 10 mM Tris-HCl pH 8.3, 1 mM EDTA pH 9.0, 1% Triton X-100), vortexed, incubated for 15 min at 95°C, cooled to room temperature, spun for 10 min at 13,000 × *g*, and the lysate was transferred to a clean tube. For the broths, 200 µL of the broth was spun for 3 min at 13,000 × *g* followed by a 1 mL wash of Tris buffer pH 7.4. The suspension was vortexed and spun at 13,000 × *g* for 3 min, and the supernatant was removed. An aliquot of 200 µL of rapid lysis buffer was added to the pellet, and the mixture was heated for 15 min at 95°C. After the heating step, the cell suspension was cooled to room temperature, spun for 10 min at 13,000 × *g*, and the lysate was transferred to a clean tube. PCR primers, probes, and cycling conditions were previously reported (23).

All isolates were sequenced as per PulseNet International protocols at the PulseNet-certified ProvLab North (Edmonton, AB), and ECTyper (24) was used to determine the serotype.

### Additional study details

Specimens were excluded from the study if they were not BD Max *stx* PCR-positive or if the above procedures were not fully followed due to technical or operational error.

In 2023, a large STEC O157 outbreak occurred in the Calgary Health Zone, resulting in 359 laboratory-confirmed cases (25). Because of the increased demand on the diagnostic and public health laboratory during this outbreak, the study protocol was not followed. Specimens collected during this period were excluded from this study.

Statistical analysis was done using GraphPad Prism 10.1 (Version 10.3.1 [464], 21 August 2024). The 95% confidence intervals (95% CI) were calculated using the Wilson/Brown method.

## RESULTS

**Fig 2:**
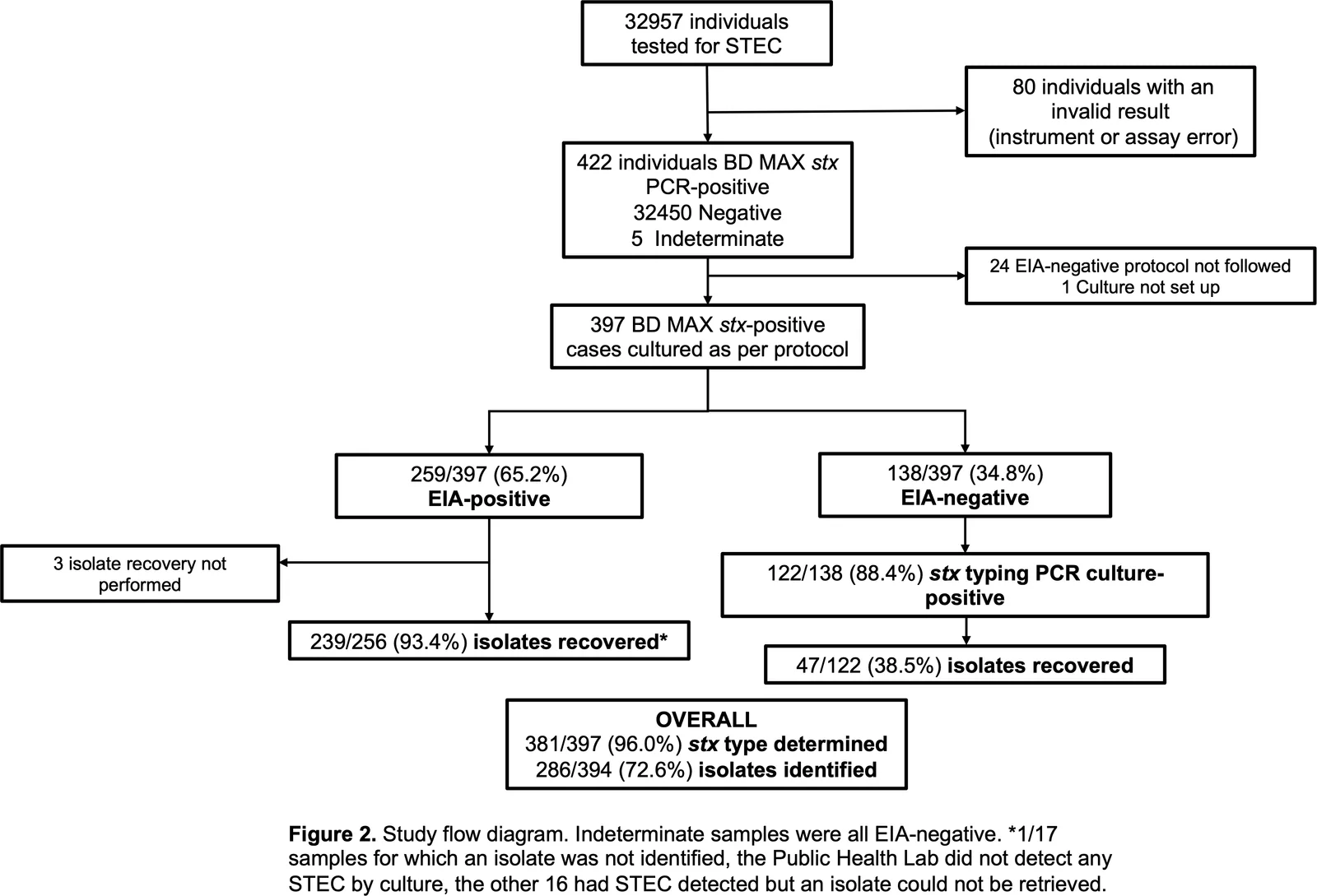
Study flow diagram. Indeterminate samples were all EIA-negative. *1/17 samples for which an isolate was not identified, the Public Health Lab did not detect any STEC by culture; the other 16 had STEC detected but an isolate could not be retrieved.

During the study period, 397 BD Max *stx*-positive cases were cultured as per protocol. Of these, 259 (65.2%) had Stx types detected in culture by EIA (Fig. 2). Of the 138 EIA-negative cultures, *stx* typing PCR on culture media/broth was positive in 122 (88.4%). Combining EIA and typing PCR culture methods, the *stx* type was identified in 96.0% of BD Max *stx* PCR-positive cases.

Strains that had *stx*1 only were more common in the cases with EIA-positive cultures, with 66.4% of cases that were EIA-positive by culture producing Stx1 only (*n* = 172), 18.5% producing Stx2 only (*n* = 48), and 15.1% producing both Stx1 and Stx2 (*n* = 39) (Table 1). In contrast, *stx*2 was more commonly detected in EIA-negative cultures, with 45.9% of typing PCR-positive cultures being positive for only *stx2* (*n* = 56), followed by only *stx*1 (35.2%, *n* = 43), then both *stx1* and *stx2* (18.9%, *n* = 23). Importantly, the proportion of infections with *stx*2 (with or without *stx*1) was significantly greater in the EIA-negative group than the EIA-positive group (64.8% compared to 33.9%, *P* < 0.0001, Fisher’s exact test).

**TABLE 1:** *stx* types detected by EIA-directed culture vs typing PCR-directed culture from BD Max *stx* PCR-positive feces^*a*^

Shiga toxin types	Culture EIA-positive (*n* = 259)	Culture EIA-negative, typing PCR-positive (*n* = 122)
1 only	172 (66.4%)	43 (35.2%)
2 only	48 (18.5%)	56 (45.9%)
1 and 2	39 (15.1%)	23 (18.9%)
Total cases with Shiga toxin 1	211 (81.5%)	66 (54.1%)
Total cases with Shiga toxin 2	87 (33.6%)	79 (64.8%)

^*a*^ Of 397 BD max *stx-*positive cases with culture done as per protocol, 16 (4.03%) had no typing result, 259 had a Stx result from the EIA, and 122 EIA-negative had a *stx* result from the typing PCR. Total cases with Shiga toxin 1 = those with *stx*1 only or *stx*1 and 2. Total cases with Shiga toxin 2 = those with *stx*2 only or *stx*1 and 2.

For cases with EIA-positive cultures, the Shiga toxin type was also confirmed when an isolate was recovered at the ProvLab using *stx* typing PCR. Typing results were discrepant in 10 instances (Table S2).

In the culture EIA-positive cases, isolate retrieval for typing was successful in 93.4% (*n* = 239 of 256, in three cases, isolate recovery was not performed) (Fig. 2). An isolate was recovered in an additional 47 of the 122 typing PCR-positive cases (38.5%). Using *stx* typing PCR-guided culture thereby increased the total isolate recovery for purposes of serotyping and whole-genome MLST to 72.6%. Using EIA alone to determine which media to recover an isolate from would have resulted in an isolate being recovered by only 60.6% of the specimens.

In addition to detecting a greater proportion of cases caused by *stx*2-positive organisms and increasing recovery of isolates for typing, our results demonstrated that cases with some serotypes were less likely to have cultures that were EIA-positive (Table 2; Table S3 for a complete list of detected serotypes). *E. coli* O157:H7 represented 19.2% of all STEC isolated and serotyped in the study period. Notably, 46 of 48 *E. coli* O157:H7 were from cases with EIA-positive cultures, and only two were identified by *stx* typing PCR-positive cultures. Similar discrepancies were noted with other common serotypes being more frequently identified from cases with EIA-positive cultures: O26:H11 (38 of 39), O111:H8 (32 of 33), O103:H2 (31 of 32), and O151/O118:H2 (12 of 13). Conversely, certain serotypes were more likely to be detected by typing PCR-guided culture, including *E. coli* O166:H15 (4/4 isolates detected only by *stx* typing PCR-guided culture) and *E. coli* O91:H14 (4/6 cases detected by *stx* typing PCR-guided culture).

**TABLE 2:** Most common STEC serotypes detected using EIA-guided culture vs typing PCR-guided culture (percent of total isolates serotyped)

Serotype	Culture EIA-positive	Culture EIA-negative, typing PCR-positive
*E. coli* O157:H7	46 (19.3%)	2 (4.3%)
*E. coli* O26:H11	38 (15.9%)	1 (2.1%)
*E. coli* O111:H8	32 (13.4%)	1 (2.1%)
*E. coli* O103:H2	31 (13.0%)	1 (2.1%)^*a*^
*E. coli* O151/O118:H2	12 (5.0%)	1 (2.1%)
*E. coli* O166:H15	0	4 (8.5%)
*E. coli* O91:H14	2 (0.8%)	4 (8.5%)
*E. coli* O76:H19	5 (2.1%)	2 (4.3%)
Other	73 (30.5%)	31 (66.0%)
Total isolates	239	47

^*a*^ Co-infection with *E. coli* O22:H8.

To establish the highest-yield culture media in the EIA-negative cultures group, we examined their relative contribution to detecting the *stx* type by PCR and isolate recovery. The typing PCR was most frequently positive from EIA culture-negative feces planted to TSB (83.3%), then to GNB (81.8%), and finally from feces directly plated on a MAC (68.8%) (Table 3). A combination of TSB and GN broth detected the *stx* type from a greater number of specimens (86.9%) than other combinations, such as direct MAC agar and TSB (85.5%) or direct MAC agar and GN broth (84.7%). Of the 47 isolates recovered from EIA culture-negative cases, they were most frequently from the MAC agar inoculated with feces directly (29.7%, *n* = 41). In four cases, isolates were recovered following sub-culture from GN broth and in two, from TSB.

**TABLE 3:** Contribution of the different media used in typing PCR-guided culture for cases with EIA-negative cultures

Media			Number of samples	%
MAC agar	GN broth	TSB		
Positive	Positive	Positive	90	65.2%
Positive	Positive	Negative	1	0.7%
Positive	Negative	Positive	2	1.4%
Negative	Positive	Positive	18	13.0%
Positive	Negative	Negative	2	1.4%
Negative	Positive	Negative	4	2.9%
Negative	Negative	Positive	5	3.6%
Negative	Negative	Negative	16	11.6%
Total			138	

Picking 15 colonies from a MAC agar is labor-intensive; however, it does result in a higher number of isolates recovered. In 37 cases of the EIA-negative group where an isolate was recovered, the number of colonies testing positive was documented. In 10 of these, 1–2 of the 15 colonies were positive; in another 8 cases, only 5 colonies were positive; and the remaining 19 had more than 5 colonies positive. In the EIA-positive culture group, data on the number of colony picks that were PCR-positive on the MAC or CHROMagar STEC were not collected.

**Fig 3:**
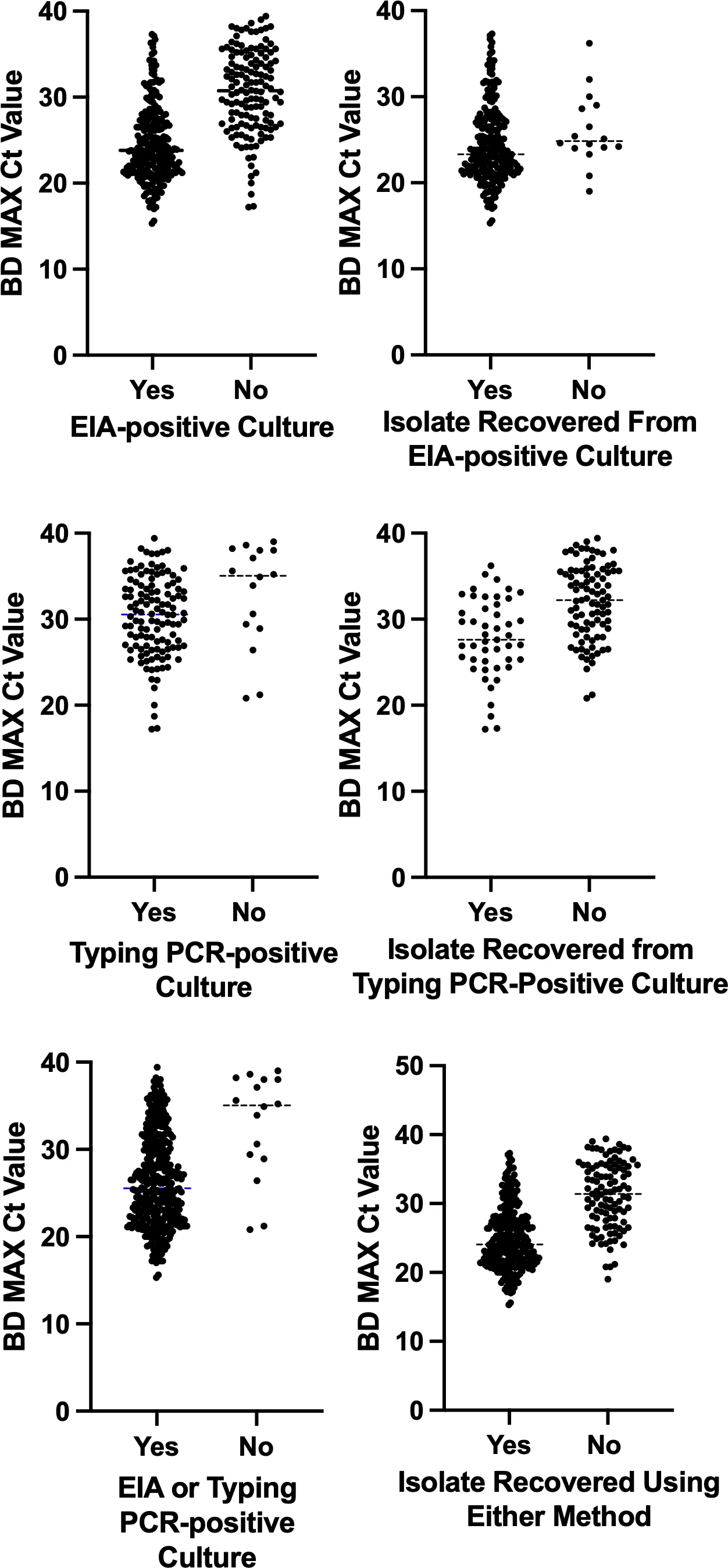
BD Max Ct value distributions for Shiga toxin (*stx*)-positive specimens in which culture was Stx EIA-positive or *stx* typing PCR-positive, or isolates were recovered from. Hashed lines on violin plots represent median and quartiles. Line on dot plot represents the median.

Median BD Max *stx* Ct values were lower when cultures were EIA-positive compared to when EIA-negative (Fig. 3; median 23.8 vs 30.8, 95th percentiles 33.6 vs 38.0, maximum 37.3 vs 39.4) (*P* < 0.0001, Wilcoxon signed-rank test). Statistically significant lower Ct values were also observed for specimens with a *stx* type detected by either culture method (EIA or typing PCR), compared to no subtype identified (median 25.6 vs 35.1, 95th percentiles 35.8 vs 39.0, maximums 39.4 vs 39.0) (Fig. 3) (*P* < 0.0001). Ct values were also lower for cases with an isolate recovered compared to none recovered (median 24.1 vs 31.4, 95th percentiles 33.6 vs 38.1, maximums 37.3 vs 39.4). Area under the receiver operating curve for BD Max Ct values was 0.822 (95% CI: 0.778–0.866) for EIA-positive vs EIA-negative, 0.819 (95% CI: 0.774–0.865) for isolate vs no isolate recovered, and 0.790 (95% CI: 0.654–0.927) for type detected by EIA or PCR vs not (all *P* < 0.0001).

## DISCUSSION

In a large, population-based post-implementation study, we successfully improved the determination of *stx* types, detection of *stx*2-possessing strains, and the recovery of STEC isolates. Our primary aim was successful, with the *stx* type identified in 96% of cases compared to only 65% if our standard culture method (EIA-guided culture) was used. Furthermore, we identified an isolate in 72% of cases using typing PCR-guided cultures and EIA compared to 61% using EIA alone. These findings demonstrate that using EIA on cultured feces to type Stx and triage specimens for isolate recovery is insufficient. Supporting the APHL recommendations on STEC culture (5), these findings provide further evidence that laboratories could consider PCR-based identification of STEC isolates in culture.

One of the most striking findings of this study was that relying on EIA for Stx typing failed to detect 47.6% of infections with STEC carrying the *stx2* gene. Therefore, relying on the *QUIK* CHEK to determine the *stx* subtype would miss clinically relevant information. We hypothesize that this is most likely due to the lower sensitivity of the EIA for Stx2 compared to Stx1, or to differences in gene expression levels. Differential growth of certain strains may have also contributed, resulting in higher or lower organism load. This is supported by our observation that O157:H7 isolates were readily detected using EIA, while some non-O157 strains were not. STEC O157:H7 has historically been the most significant serotype, and many culture techniques have been optimized for its recovery. One additional caveat is that we did not determine whether the *stx*2 gene-positive strains could produce Stx2. Regardless, because Stx2-producing strains are more likely to cause extraintestinal complications like HUS, this observation is of great importance. This algorithm identified previously unknown *stx* typing information to clinicians and public health, while improving our understanding of the prevalence of *stx*2 in our setting, and warrants further investigation.

Based on our findings, *stx*2 detection by nucleic acid tests should be used for maximum impact on clinical management. Although we ultimately detected the *stx* type in almost all cases, a quicker turnaround time (TAT) than that provided by our algorithm would greatly facilitate clinical and public health management. In our setting, the average collect-to-received TAT for BD Max *stx*-positive results was 2.3 days (testing run daily), 2.9 days for an EIA-positive culture result, and 6.2 days for the PCR-guided culture result (run two to three times per week). The average collect-to-received time was 1 day, as most are from the community. Using our algorithm, which paired diagnostic and public health laboratory services, we provided a rapid and clinically actionable Stx typing result in 60% of cases while still obtaining a meaningful *stx* typing result in the remaining cases within a timeframe that may allow detection of early laboratory abnormalities suggestive of HUS (26).

To achieve shorter turnaround times, it would require integrating *stx*1 and *stx2* differentiation into the primary screening PCR, by performing a reflex direct-on-specimen *stx* typing PCR at the frontline diagnostic lab, or running the PCR-guided culture testing daily at the public health laboratory. Alternatively, typing PCR could be performed on a sweep of colonies from agar (CHROMagar and/or MAC) with or without performing PCR on enrichment broth, the latter sacrificing isolate yield. The added benefit of this approach is that the sweep of colonies would provide a *stx* typing result while simultaneously determining culture positivity and identifying samples with a high likelihood of isolate recovery. At the time of writing this manuscript, we are aware of two gastroenteritis panel assays that distinguish between the *stx* types on the primary specimen that were approved by the FDA or Health Canada after our study was concluded (Xpert GI Panel, Cepheid, and QIAstat-Dx Gastrointestinal Panel 2, Qiagen N.V.) and none from isolates/culture. Due to greater cost and additional targets on these panels, these platforms may not be appropriate in many settings; this leaves the options of using lab-developed tests or commercial kits that have not been approved by a regulatory agency.

The ability to run an additional PCR (particularly a lab-developed test) on the fecal specimen or on growth from multiple media may be prohibitive for many labs because of cost, reimbursement, expertise, regulatory, and labor constraints (27). This would therefore leave the identification of *stx*1 and 2 and/or recovery of the isolates to public health laboratories, provided funding is available. In such a model, the TAT, and therefore clinical usefulness, would depend on how well diagnostic and public health laboratories are integrated and how frequently the various testing steps are performed. One of the most human-resources-limiting steps is the PCR-directed culture, which we estimate to take 1.5–3 h per sample depending on the number of colony picks and whether subbing from broth is needed. Despite these hurdles, our finding that rapid EIA-based testing misses many *stx*2 cases indicates that investment into quicker *stx*2 processes, such as direct feces typing PCR or faster turnaround time for PCR-guided culture, would be worthwhile. The clinical benefit of earlier detection of *stx*2 and targeted intervention to prevent HUS or detect the development of HUS earlier could justify the additional effort and expense. The economic benefit is likely significant, given that preventing dialysis would result in long-term health care system savings.

Culture positivity and isolate recovery were more likely with a lower BD Max Ct value, as 95% of samples with an isolate recovered had a BD Max Ct less than 34. Therefore, laboratory workload could be reduced by not attempting to culture those samples with a Ct more than 34. However, it is important to note that the BD Max is FDA- and Health Canada-approved as a qualitative assay; fecal specimens are heterogeneous, and the reproducibility of Ct values has not been established, so caution should be taken when interpreting Ct values in this way.

Some labs have reported picking a maximum of 10–100 colonies to improve recovery (Table S1); the upper end is not feasible for many laboratories (frontline and public health) due to human and financial resource limitations. We selected 15 colonies from the MAC agar based on previous experience at ProvLab, and our data showed that picking at least this number of colonies was necessary, as in many cases, only 1–3 *stx-*positive colonies were detected out of a total of 15 picked. Increased workload may be why the APHL suggested that labs perform PCR on five colonies, but this study and others (Table S1) show that this is suboptimal (5). The protocol used in our procedure minimized labor by focusing on the MAC agar sweep and broths with *stx* typing PCR Ct values below 32 to triage cases from which an isolate was sought. Another factor affecting isolate recovery that we have anecdotally observed over many years of searching for STEC isolates is that a technologist’s expertise can also influence the recovery. Clearly, further work on identifying the optimal number of colonies to pick and the risk-benefit analysis from the public health perspective is needed.

Our study has clinical and public health implications. First and foremost, providing information about the *stx* type can help identify the risk of developing HUS, justify closer follow-up or intervention (e.g., intravenous fluids or HUS-monitoring blood work), and triage case investigations. Second, we think these results will improve clinicians’ and public health practitioners’ trust in PCR results. Our study clearly demonstrates that STEC can be frequently isolated in culture when PCR-positive, even though it may be difficult to isolate with traditional culture methods. This was also demonstrated in a 2023 study from Finland, which showed that 89% of PCR-positive cases were culture-positive when they used CHROMagar Orientation agar (often used for urine cultures) and a colony-sweep STEC PCR to identify growth. Our data indicate that a viable organism is present in >95% of PCR-positive specimens when the specimen is the first submitted from a case; therefore, PCR results alone should be considered clinically significant. As most public health jurisdictions use culture to exclude individuals from attending sensitive situations or occupations (11), the first stool submitted for diagnosis should be considered culture-positive. Jääskeläinen et al. determined that two separately collected PCR-negative fecal samples suffice to demonstrate clearance of culturable organisms (13), and Besser et al. also concluded PCR could be used for this purpose (11).

Our study has many strengths, including a large number of cases, representation from community and acute care patients, representing all ages, and specimens collected over two summers when STEC disease peaks (10). One main disadvantage of this study was that the antigen-guided culture and the typing PCR-guided culture were not set up simultaneously, so *stx* typing and isolate recovery could have been greater than reported herein. However, specimens were sent to the ProvLab and plated to media within 24–72 h of the EIA-guided culture result, a time frame unlikely to affect STEC recovery, as STEC is stable in raw feces (no transport media) for at least 7 days (28). For practical reasons, we also did not assess recovery of isolates from media when the Ct value was greater than 32 on MAC agar, and we limited our colony picks to 15 from MAC. We may have recovered more isolates if more resource-intensive workup had been done.

In conclusion, we present methods to improve the recovery of STEC isolates and determine if *stx*1 and *stx2* are present. While doing so, we demonstrated high agreement between culture and PCR. We also demonstrated that the Shiga Toxin *QUIK CHEK* missed many *stx*2-carrying strains even when performed on cultures, and that PCR-positive specimens are likely to represent specimens with a viable organism. This work could enhance our ability to manage STEC infections, prevent HUS, and manage STEC outbreaks.

## SUPPLEMENTAL MATERIAL

Figure S1 jcm.00429-26-s0001.tiff Isolate recovery processes.

Supplemental tables jcm.00429-26-s0002.docx Tables S1 to S3.

ASM does not own the copyrights to Supplemental Material that may be linked to, or accessed through, an article. The authors have granted ASM a non-exclusive, world-wide license to publish the Supplemental Material files. Please contact the corresponding author directly for reuse.
