# Supplementary material for: Improving isolate recovery and identification of the Shiga toxin type in Shiga toxin nucleic acid test-positive feces
Source: J Clin Microbiol. 2026 Aug 14;64(9):e00429-26. doi: 10.1128/jcm.00429-26 (PMC13556236; doi:10.1128/jcm.00429-26)
Supplement: Supplementary file 1 [file jcm.00429-26-s0002.docx]

Supplementary Table 1. Different methods of culture (Cx) recovery from Shiga toxin-producing *E. coli* STEC PCR-positive fecal specimens

| Study | Initial Screen | STEC Cx Method | Numbers tested and positive | Cx positivity | Isolation % (#) |
| --- | --- | --- | --- | --- | --- |
| Jaaskelainen 2023 | LDT PCR on feces | BD™ CHROMagar™ Orientation, sweep using LDT PCR Number of colonies picked not reported | 15 365 samples;  13 528 patients;  236 (1.7%) cases | 89% (211/236) | 58.9% (139/236) |
| Lefterova 2013 | LDT PCR on GNB | GNB planted to O157 CHROMAgar. If negative (STEC detection method not described) PCR on broth and "conventional media”. | 4900 samples;  65 (1.32%) cases | Unclear | 66.2% (43/65) |
| Vallieres 2013 | LDT PCR, Premier and Immunocard EIA on MAC broth + SMAC | MAC broth to MAC plate. PCR on up to 100 colonies. | 632 samples, 430 patients;  21 (1.6%) positive  12 (2.8%) cases | 100% from MAC (primary screen); 71.4% (15/21) by SMAC, Premier or Immunocard | 76.2% 16/21 of stools |
| Jenkins 2020 | LDT PCR on TSB | Stool planted to MAC and CHROMagar. PCR on 10 colonies from MAC and one colony from CHROMagar. | sample # unknown;  292 positives | 70% (205/292) * | 36.3% (106/292) |
| Gerritzen 2011  (STEC O104:H4 outbreak) | LDT PCR on feces | All stools also planted to Vero cell Cx + TSB. ProSpect EIA on agars from TSB subculture. | 754 specimens  127 (16.8%) positive by any assay;  122 (16.2%) PCR-positive | 91.8 % (112/122) PCR-positive by EIA, plates, or Vero cell Cx** | Unclear |
| Penzel 2020 | BD Max PCR on feces | Stool to Brilliance *E. coli*/coliform chromogenic agar (Oxoid) and GNB. Colony or broth PCR using Ridagene EHEC/EPEC PCR. Easyplex LAMP on GNB. | 4996 specimens;  58 (1.2%) positive | 46.6% (27/58) | 20.7% (12/58)  11/12 isolates were *E. coli* O157 |
| Qin 2015 | LDT PCR + ImmunoCard on MAC broth | MAC broths to SMAC, MAC, and Spectra urinary tract infection (UTI) agar. STEC detected by O157 sera for SMAC. LDT PCR for others. PCR done on 5-6 colonies initially, up to 30. | 2,386 specimens;  53 (2.2%) positive | 100% from MAC broth (primary screen). 54.7% (29/53) using EIA. | 62.3% (33/53) |
| Bai 2018 | LDT PCR on blood agar | Stools to a MAC plate and LDT PCR on 10 colonies. *** | 14550 specimens;  175 (1.2%) positive | 100% but primary PCR on blood agar | 43% (75/175) |
| Shah 2024 | Methods used at U.S.A FoodNet sites | Various methods. Not described. | 3348 CIDT-positive cases, 2516 (75.1%) were cultured | Not reported | 56.5% (1422/2516) |
| Delahoy 2023 |  |  | 2,882 CIDT-positive cases, 2,401 (83%) were cultured | Not reported | 54% (1297/2401) |
| Vishram 2021 | As per referring lab  2014-2018 | Methods as per DOI: 10.3389/fmicb.2016.00258  United Kingdom National Enhanced Surveillance System | 5844 cases;  2579 non-O157 | Not reported | 62% (964/2579) Reported for non-O157 only |

LDT=Lab-developed test; PCR=polymerase chain reaction; GNB=Gram-negative broth; EIA=Enzyme immunoassay; STEC= Shiga toxin-producing *E. coli*; MAC=MacConkey Agar; SMAC=Sorbitol-MacConkey Agar; TSB= Trypticase soy broth. *51.7% on MAC and CHROMagar (106), 48.3% MAC only (n=99) **121/122 PCR-positive samples were *stx_2_* positive. 88.5% (108/122) of PCR-positives were positive by EIA.***Paper says SMAC used, but reference provided says MAC (Svenengsson *et al.* Clinical Infectious Diseases 2000 DOI: [10.1086/313770](https://doi-org.ezproxy.lib.ucalgary.ca/10.1086/313770))

Supplementary Table 2. Discordant Shiga toxin enzyme immunoassay (EIA) positive results compared to the reference lab (Public Health Laboratory) Shiga toxin subtype PCR result

| Number of Samples | EIA Stx Result | | ProvLab *stx_1/2_* Result | |
| --- | --- | --- | --- | --- |
|  | Stx1 | Stx2 | *stx_1_* | *stx_2_* |
| 7 | Positive | Negative | Positive | Positive |
| 2 | Negative | Positive | Positive | Positive |
| 1 | Positive | Positive | Positive | Negative |

Two Stx 1&2 EIA-positives did not have PCR done, but were O157 agglutination positive, so presumed concordant. One Stx1 EIA-positive did not have PCR done but was EIA-positive for Stx 1 at ProvLab and considered concordant.

Supplementary Table 3. Serotypes of isolates from A) Enzyme immunoassay-guided culture and B) Typing PCR-guided culture.

| Serotype | Number of Isolates | % |
| --- | --- | --- |
| *Escherichia coli* O157:H7 | 46 | 19.2% |
| *E. coli* O26:H11 | 38 | 15.8% |
| *E. coli* O111:H8 | 32 | 13.3% |
| *E. coli* O103:H2 | 31 | 12.9% |
| *E. coli* O151/O118:H2 | 12 | 5.0% |
| *E. coli* O121:H19 | 9 | 3.8% |
| *E. coli* O123/O186:H2 | 9 | 3.8% |
| *E. coli* O145:H Nonmotile | 7 | 2.9% |
| *E. coli* O5:H9 | 5 | 2.1% |
| *E. coli* O71:H11 | 5 | 2.1% |
| *E. coli* O76:H19 | 5 | 2.1% |
| *E. coli* O103:H25 | 4 | 1.7% |
| *E. coli* O17:H45 | 3 | 1.3% |
| *E. coli* O103:H11 | 2 | 0.8% |
| *E. coli* O151/O118:H16 | 2 | 0.8% |
| *E. coli* O177:H25 | 2 | 0.8% |
| *E. coli* O71:H8 | 2 | 0.8% |
| *E. coli* O91:H14 | 2 | 0.8% |
| *E. coli* O91:H14 | 1 | 0.4% |
| *E. coli* O Undetermined:H45 | 1 | 0.4% |
| *E. coli* O100:H20 | 1 | 0.4% |
| *E. coli* O108:H25 | 1 | 0.4% |
| *E. coli* O112:H21 | 1 | 0.4% |
| *E. coli* O113:H21 | 1 | 0.4% |
| *E. coli* O116:H49 | 1 | 0.4% |
| *E. coli* O136:H12 | 1 | 0.4% |
| *E. coli* O146:H21 | 1 | 0.4% |
| *E. coli* O153/O178:H19 | 1 | 0.4% |
| *E. coli* O153/O178:H7 | 1 | 0.4% |
| *E. coli* O156:H25 | 1 | 0.4% |
| *E. coli* O156:H7 | 1 | 0.4% |
| *E. coli* O158:H Undetermined | 1 | 0.4% |
| *E. coli* O174:H21 | 1 | 0.4% |
| *E. coli* O5:H19 | 1 | 0.4% |
| *E. coli* O55:H12 | 1 | 0.4% |
| *E. coli* O69:H11 | 1 | 0.4% |
| *E. coli* O76:H38 | 1 | 0.4% |
| *E. coli* O84:H2 | 1 | 0.4% |
| *E. coli* O92:H33 | 1 | 0.4% |
| *E. coli* O93:H46 | 1 | 0.4% |
| *E. coli* O98:H21 | 1 | 0.4% |
| Grand Total | 239 | 100.0% |

B.

| Serotype | # of isolates | % |
| --- | --- | --- |
| *E. coli* O166:H15 | 4 | 8.5% |
| *E. coli* O91:H14 | 4 | 8.5% |
| *E. coli* O109:H5 | 2 | 4.3% |
| *E. coli* O36:H42 | 2 | 4.3% |
| *E. coli* O5:H19 | 2 | 4.3% |
| *E. coli* O76:H19 | 2 | 4.3% |
| *E. coli* O9:H7 | 1 | 2.1% |
| *E. coli* O157:H7 | 2 | 4.3% |
| *E. coli* O Undetermined:H2 | 1 | 2.1% |
| *E. coli* O103:H25 | 1 | 2.1% |
| *E. coli* O108:H21 | 1 | 2.1% |
| *E. coli* O111:H8 | 1 | 2.1% |
| *E. coli* O117/O107:H7 | 1 | 2.1% |
| *E. coli* O117:H7 | 1 | 2.1% |
| *E. coli* O119:H2 | 1 | 2.1% |
| *E. coli* O121:H19 | 1 | 2.1% |
| *E. coli* O140:H21 | 1 | 2.1% |
| *E. coli* O146:H21 | 1 | 2.1% |
| *E. coli* O156:H14 | 1 | 2.1% |
| *E. coli* O168:H8 | 1 | 2.1% |
| *E. coli* O175:H5 | 1 | 2.1% |
| *E. coli* O183:H18 | 1 | 2.1% |
| *E. coli* O2/O50:H7 | 1 | 2.1% |
| *E. coli* O22:H1 | 1 | 2.1% |
| *E. coli* O22:H8; O103:H2 coinfection \| | 1 | 2.1% |
| *E. coli* O25:H1 | 1 | 2.1% |
| *E. coli* O26:H11 | 1 | 2.1% |
| *E. coli* O42/O28:H25 | 1 | 2.1% |
| *E. coli* O51:H41 | 1 | 2.1% |
| *E. coli* O8:H17 | 1 | 2.1% |
| *E. coli* O8:H19 | 1 | 2.1% |
| *E. coli* O8:H28 | 1 | 2.1% |
| *E. coli* O95:H7 | 1 | 2.1% |
| *E. coli* O128:H2 | 1 | 2.1% |
| *E. coli* O151/O118:H2 | 1 | 2.1% |
| *E. coli* O1:H7 | 1 | 2.1% |
| Grand Total | 47 | 100.0% |
